# *Scedosporium apiospermum* Infective Endocarditis With Brain Abscesses in a Lung Transplant Recipient: Review of the Literature and Evaluating the Use of Next‐Generation Sequencing

**DOI:** 10.1155/crdi/8041837

**Published:** 2026-05-17

**Authors:** Emily Wong, Julie England, Vidya Jagadeesan

**Affiliations:** ^1^ Division of Infectious Diseases, University of Texas Southwestern Medical Center, Dallas, Texas, USA, utsouthwestern.edu

**Keywords:** fungal endocarditis, Karius, metagenomic next-generation sequencing, *Scedosporium apiospermum*, solid-organ transplant, voriconazole

## Abstract

*Scedosporium apiospermum* is an emerging cause of invasive mold infection in immunocompromised hosts, often with central nervous system involvement and limited susceptibility to amphotericin B. We describe a 36‐year‐old lung transplant recipient who presented with fever, meningismus, and multiple enhancing brain lesions nine months post‐transplant. Cerebrospinal fluid studies, including metagenomic next‐generation sequencing (mNGS), were negative. Cardiac imaging revealed a pedunculated right ventricular septal mass, and plasma cell‐free DNA (cfDNA) testing (Karius) identified *S. apiospermum*. Subsequent brain biopsy and thrombectomy confirmed the diagnosis by histopathology and culture. Following surgical removal of the cardiac mass and treatment with voriconazole, the patient improved with near resolution of brain lesions. This case highlights disseminated *S. apiospermum* endocarditis diagnosed by plasma cfDNA despite negative CSF mNGS, underscoring that site‐specific mNGS may be falsely negative in compartmentalized infections. Plasma cfDNA testing can complement conventional and tissue‐based diagnostics for early detection of disseminated mold infections in transplant recipients.

## 1. Introduction

*Scedosporium apiospermum* (*S. apiospermum)* is an emerging cause of invasive mold infection in immunocompromised hosts, often with central nervous system (CNS) involvement and limited susceptibility to amphotericin B. Fungal endocarditis carries a high mortality rate, particularly in immunocompromised hosts. Here, we present a case of *S. apiospermum* endocarditis, with ventricular septal vegetation and dissemination to the brain, successfully treated with voriconazole and thrombectomy. We will then discuss the utility of next‐generation sequencing as a diagnostic strategy in populations at high risk for invasive mold infection. Given the growing population of immunocompromised patients and the expanding use of hypothesis‐free diagnostics, understanding the clinical spectrum and diagnostic pitfalls of *Scedosporium* infections has important implications for early recognition and management. This is especially true in solid organ transplant (SOT) recipients, where atypical presentations need to be recognized [1].

## 2. Case Presentation

A 36‐year‐old female with a past medical history of systemic lupus erythematosus (SLE), antiphospholipid syndrome, and interstitial lung disease underwent bilateral sequential lung transplantation. Her post‐transplant course was complicated by Grade 3 primary graft dysfunction requiring veno‐venous extracorporeal membrane oxygenation (VV ECMO) for 14 days. One month post‐transplant, she developed donor‐specific antibodies (DSA) and received one dose of rituximab, followed by monthly carfilzomib and intravenous immune globulin (IVIG). Two months post‐transplant, she developed sternal wound dehiscence requiring debridement and chest closure. Posaconazole was administered as primary antifungal prophylaxis due to pretransplant *Aspergillus terreus* colonization and continued for approximately 3 months post‐transplant, with a documented therapeutic level of 1.07 μg/mL.

Nine months post‐transplant (6 months after discontinuation of posaconazole), she presented with acute onset headache, neck stiffness, fever, and vomiting beginning on the day of admission. She lived with her parents in an apartment in North Texas with nearby construction exposure. She denied sexual activity, substance use, recent travel, or outdoor exposures. Maintenance immunosuppression included mycophenolate 1000 mg twice daily, prednisone 10 mg daily, and tacrolimus 2 mg every morning and 1.5 mg every evening. Her last dose of carfilzomib was 3 months ago, and her last dose of IVIG was 1 month ago. Prior to transplant, the patient was on hydroxychloroquine, prednisone, and mycophenolate for SLE.

Vital signs were notable for a fever of 100.5°F and heart rate of 118. The patient was in severe pain due to a headache and unable to tolerate passive flexion of her neck. There was thrush on oral exam. Magnetic resonance imaging (MRI) of the brain showed enhancing foci in the right caudate nucleus, right external capsule, right occipital lobe, and new right frontal horn ependymal enhancement. A lumbar puncture was performed with an opening pressure of 20 cm H_2_O. Cerebrospinal fluid (CSF) had 98 nucleated cells, with 64% monocytes and 21% lymphocytes. CSF bacterial, mycobacterial, and fungal cultures were negative. Other negative CSF tests included cryptococcal antigen, West Nile virus (WNV) IgG/IgM and polymerase chain reaction (PCR), herpes simplex virus 1 and 2 PCR, varicella zoster PCR, toxoplasma PCR, Epstein–Barr virus PCR, enterovirus PCR, and histoplasma antigen. CSF next‐generation metagenomic sequencing (mNGS) was negative.

While awaiting results, empiric intravenous vancomycin, ceftriaxone, and ampicillin were started. Interestingly, within several days, the patient’s fever resolved. Her headaches improved but continued to wax and wane. Her thrush was also treated with clotrimazole troches with clinical resolution.

Transesophageal echocardiogram (TEE) was obtained to evaluate for embolic phenomena as the source of brain lesions. This demonstrated a pedunculated mobile mass (1.5 × 0.8 cm) in the right ventricle, attached to the septal wall. On cardiac MRI, the radiographic impression noted the mass was suggestive of thrombus, but the high signal intensity on the T2‐weighted image was atypical for clot appearance.

**FIGURE 1:**
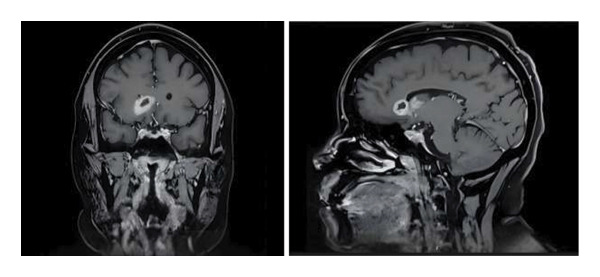
Repeat T2 FLAIR brain MRI with and without contrast—showing worsening rim—enhancing lesions within the right anterior frontal lobe/corpus callosum genu.

Given the unclear diagnosis with ongoing headaches, a brain MRI was repeated after 2 weeks of empiric antibiotic treatment. Unfortunately, this demonstrated a worsening rim‐enhancing lesion in the right frontal lobe and several new areas of enhancement in the right cerebral hemisphere (Figure 1). At this time, a Karius assay resulted positive for *S. apiospermum* at 63 molecules per microliter (MPM). Empiric voriconazole was initiated. Computed tomography (CT) scan of the chest and sinuses did not show radiographic evidence of mold infection.

Due to progression on MRI and detection of *S. apiospermum* on Karius, a stereotactic brain biopsy was pursued. Pathology showed clusters of septate hyphae with positive Grocott methenamine silver (GMS) stain. Tissue cultures grew *S. apiospermum* (Figure 2(a)). Subsequently, interventional radiology performed a fluoroscopic‐guided suction thrombectomy of the right ventricular pedunculated mass, which rapidly grew *Scedosporium* species on culture and showed numerous clusters of fungal hyphae on histopathology (Figure 2(b)). Antifungal susceptibility testing demonstrated minimum inhibitory concentrations (MICs) of 0.5 μg/mL for voriconazole, posaconazole, and itraconazole; 2 μg/mL for isavuconazole; and 16 μg/mL for amphotericin B. The patient was continued on voriconazole monotherapy after discharge, with repeat brain MRI at 4 months showing near resolution of brain lesions.

FIGURE 2(a) Right frontal lobe biopsy tissue cultures grew *Scedosporium apiospermum*, shown with lactophenol cotton blue stain. (b) Right ventricular mass pathology demonstrated numerous clusters of fungal hyphae. Courtesy of Dr. Luis De Las Casas, professor of pathology, University of Texas Southwestern Medical Center.

**Figure:**
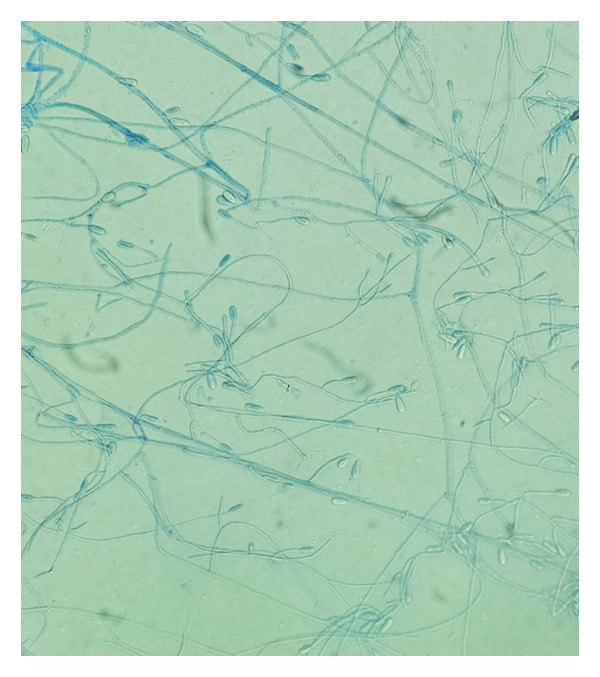
(a)

**Figure:**
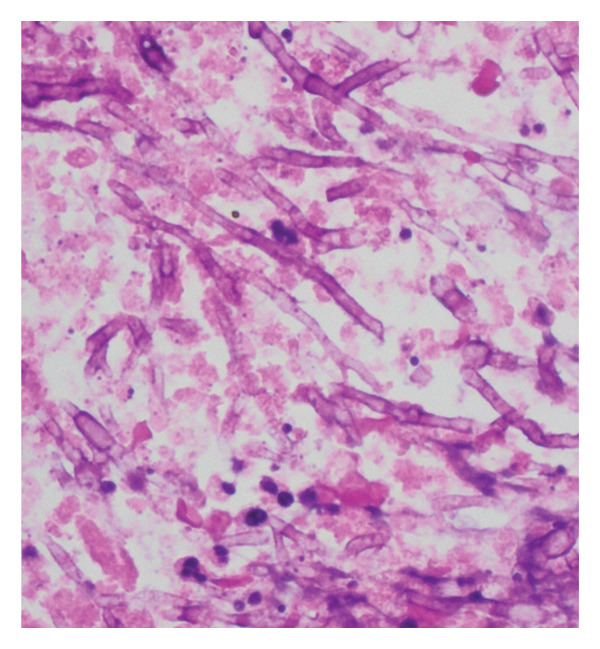
(b)

## 3. Discussion

*S. apiospermum* is a saprophytic environmental mold found in soil, sewage, and decaying vegetation. It is increasingly recognized as an opportunistic pathogen with high rates of dissemination and mortality, particularly among immunocompromised hosts [2]. Prior studies have demonstrated that *S. apiospermum* and *Scedosporium (now Lomentospora) prolificans* account for approximately 25% of non‐*Aspergillus* mold infections in SOT recipients, with higher rates of dissemination, CNS involvement, and mortality compared to *Aspergillus* species [3]. Diagnosis can be challenging because *Scedosporium* shares histopathologic features with other hyaline molds such as *Aspergillus*, showing irregular, branching, and septate hyphae. Although obovoid conidia may help distinguish Scedosporium, they are not consistently observed [4]. Differentiation is critical given marked differences in antifungal susceptibility: *S. apiospermum* exhibits variable resistance to amphotericin B, and both clinical and in vitro data support voriconazole as preferred first‐line therapy. In vitro studies show that Voriconazole consistently has the lowest MICs in *S. apiospermum* isolates, with resistance being a rare occurrence [2, 5]. Isavuconazole and posaconazole may demonstrate variable activity, but antimicrobial susceptibility testing remains essential given heterogeneous MICs with triazoles other than voriconazole [5]. Current guidelines recommend susceptibility testing when available to help guide antifungal selection, particularly given the intrinsic resistance of *Scedosporium* species to amphotericin B and variable susceptibility to other agents. In this case, a voriconazole MIC of 0.5 μg/mL supported the use of voriconazole as targeted therapy, which was associated with a favorable clinical response. These recommendations are reflected in the consensus guidelines from the European Confederation of Medical Mycology (ECMM), International Society for Human and Animal Mycology (ISHAM), and American Society for Microbiology (ASM) [2, 6].

While *S. apiospermum* is increasingly recognized as a cause of disseminated infection in immunocompromised hosts, cardiac involvement is exceedingly rare [7]. Fungal endocarditis due to *Scedosporium* species has been reported only in isolated cases and often presents with embolic or CNS manifestations as the first clue to diagnosis. These infections are diagnostically challenging due to nonspecific imaging findings and frequent culture negativity.

Fungal endocarditis overall carries a high mortality rate, up to 40% in a review by Meena et al. [7]. Survival is highest with combined surgical and antifungal therapy, while immunocompromised status predicts poorer outcomes, with survival rates of only 30%. *Candida* and *Aspergillus* species account for the majority of cases (49.6% and 30%, respectively), while *Scedosporium* species represent a small but increasing proportion (3.2%), likely reflecting both improved diagnostics and expanding use of immunosuppressive therapies. Although the aortic valve is the most frequent site of fungal endocarditis, approximately 22% of cases occur at atypical locations such as the interatrial or interventricular septum [7].

Most reported cases of *S. apiospermum* endocarditis have occurred in patients with prior valve replacement, pacemakers, or implantable cardioverter‐defibrillator (ICD) devices (Table 1) [8–22]. The majority of these patients were immunocompetent, but the mortality rate remained high, with many diagnoses made postmortem [8, 11, 16, 21]. To date, only four other cases of *S. apiospermum* endocarditis have been reported in SOT recipients, underscoring the paucity of *Scedosporium* outcomes in the SOT population [9, 16, 22, 23].

**TABLE 1:** Published cases of *Scedosporium apiospermum* endocarditis.

Reference	Year	Age Gender	Risk factor	Infected sites	Therapy	Outcome
PR	2025	36F	Lung transplant	RA mass, brain abscess	VORI, thrombectomy	Survived
Jackson et al.	2024	52M	None	Myocardium, brain abscess	None	Death
Bourlond et al.	2023	52M	Heart transplant	RA mass, lungs, muscle, subcutaneous tissue	VORI, anidulafungin	Death
Ali et al.	2023	67M	Renal transplant	Myocardial wall, lungs, brain, thyroid	AmB	Death
Clement et al.	2015	70M	Heart transplant	Tricuspid valve, paratracheal mass	VORI, micafungin	Death
Foo et al.	2009	80M	Pacemaker	Pacemaker leads, RA, lung, brain	Surgery	Death
Laurini et al.	2009	78M	Pacemaker	Tricuspid valve, pacemaker leads, endocardium wall, lung abscess	Surgery	Death
Sarvat et al.	2007	58M	Pacemaker	ICD leads, tricuspid valve, lung, endophthalmitis	Surgery, valve replacement	Survived
Verghese et al.	2005	43M	Prosthetic valve	Mitral valve, systemic emboli	AmB, itraconazole, surgery	Death
O’Bryan et al.	2002	78M	None	Tricuspid valve, lungs, endophthalmitis, joints	Itraconazole	Death
Sobottka et al.	1999	18F	Polytrauma from MVC	Mitral valve, brain abscesses	Miconazole, surgery	Death
Welty et al.	1992	54F	Liver transplant	Pulmonic valve, systemic emboli and microabscesses	None	Death
Raffanti et al.	1990	53M	Advanced HIV	Mitral valve, brain, aortoiliac emboli	None	Death
Gordon et al.	1985	52M	Aortic valve replacement	Aortic valve prosthesis	Surgery	Death
Davis et al.	1980	62F	Pacemaker	Tricuspid valve, endophthalmitis, lung	None	Death
Wain et al.	1979	NR	Unknown	Unspecified endocarditis	NR	Death
Roberts et al.	1977	48M	Prosthetic mitral valve	Mitral valve prosthesis	None	Death

*Note:* M, male; F, female; VORI, voriconazole; AmB, liposomal amphotericin B.

Abbreviations: MVC, motor vehicle accident; NR, not recorded; PR, present record; RA, right atrium.

mNGS has emerged as a valuable diagnostic adjunct for immunocompromised patients at risk for invasive fungal disease. Both plasma cell‐free DNA assays (e.g., Karius) and CSF mNGS are hypothesis‐free methods capable of broad pathogen detection, but they differ in their diagnostic utility across syndromes. Plasma cfDNA testing demonstrates the greatest sensitivity for bloodstream and disseminated infections, with reported specificities > 90% and sensitivities ranging from ∼50–85% for fungal pathogens [7, 24–26]. Conversely, CSF mNGS is optimized for CNS infections, with reported specificities of 83%–99%, but variable sensitivity (60%–97%) [27, 28].

The role of mNGS in SOT recipients remains incompletely defined. Recent studies in hematopoietic stem cell transplant populations show that CSF mNGS can detect CNS pathogens, including molds, and may shorten time to diagnosis compared with conventional culture‐based methods—facilitating earlier antifungal initiation and improved outcomes [29, 30]. In transplant populations at risk for atypical pathogens, there is utility in integrating mNGS into the diagnostic algorithm to improve early pathogen identification and guide targeted therapy. However, this case highlights that negative site‐specific mNGS does not exclude infection, particularly in disseminated or compartmentalized disease. The clinical utility of mNGS depends on careful interpretation within the context of clinical presentation, imaging, and conventional laboratory data [25, 26, 31].

The discordant results in this case—positive plasma cfDNA but negative CSF mNGS—likely reflect several factors. Firstly, *Scedosporium* species are known to cause angioinvasive disease with hematogenous dissemination, which may enhance detection in plasma. Even at what may be construed as low amounts of organisms, detection of circulating cfDNA of any amount of noncommensal environmental mold would be concerning for clinically significant disease rather than colonization in the immunocompromised host. Studies have demonstrated high concordance between plasma mold cfDNA results and conventional invasive diagnostics (e.g., tissue biopsy and bronchoalveolar lavage), supporting its clinical utility for noninvasive diagnosis of invasive mold disease [25]. Lastly, with the formation of cerebral abscesses in our case, infection contained within the parenchymal space could have yielded a false negative CSF mNGS test due to limited microbial shedding into the CSF itself. Diagnostic stewardship is essential to maximize benefit and avoid misdiagnosis when using mNGS tests. False negatives may occur in low‐burden or compartmentalized disease, and false positives can result from contamination or misinterpretation of low‐level detection [25, 26, 31]. Multidisciplinary review and, when possible, additional testing for confirmation are recommended for actionable results.

## 4. Conclusion

This case highlights the diagnostic and therapeutic challenges of *S. apiospermum* infection in transplant recipients. Plasma cfDNA testing may provide an early diagnostic clue in disseminated mold infections when site‐specific mNGS and conventional cultures are negative. Clinicians should interpret these assays within the clinical and radiographic context and maintain a high index of suspicion for atypical infections in immunocompromised patients with embolic or CNS lesions.

## Funding

No funding was received for this manuscript.

## Disclosure

The study received no external funding or sponsorship, and the authors had full access to all the data and take full responsibility for the integrity of the data and the accuracy of the data analysis.

## Consent

This case report does not include factors necessitating patient consent, and all feasible steps were taken to anonymize the case and ensure confidentiality.

## Conflicts of Interest

The authors declare no conflicts of interest.

## Data Availability

The data supporting the findings of this case report, including de‐identified laboratory and imaging data, are included in the article and its supporting files. Additional clinical details are available in the electronic health record but cannot be shared publicly due to patient privacy concerns; limited data may be available from the corresponding author upon reasonable request and with appropriate institutional approvals.
